# Innate and learnt color preferences in the common green-eyed white butterfly (*Leptophobia aripa*): experimental evidence

**DOI:** 10.7717/peerj.12567

**Published:** 2021-11-29

**Authors:** Deysi Muñoz-Galicia, Citlalli Castillo-Guevara, Carlos Lara

**Affiliations:** Centro de Investigación en Ciencias Biológicas, Universidad Autónoma de Tlaxcala, San Felipe, Ixtacuixtla, Tlaxcala, Mexico

**Keywords:** Cognition, Innate color preferences, Learning, Pieridae, Visual cues

## Abstract

**Background:**

Learning abilities help animals modify their behaviors based on experience and innate sensory biases to confront environmental unpredictability. In a food acquisition context, the ability to detect, learn, and switch is fundamental in a wide range of insect species facing the ever-changing availability of their floral rewards. Here, we used an experimental approach to address the innate color preferences and learning abilities of the common green-eyed white butterfly (*Leptophobia aripa*).

**Methods:**

In Experiment 1, we conducted innate preference choice-tests to determine whether butterflies had a strong innate color preference and to evaluate whether color preferences differed depending on the array of colors offered. We faced naïve butterflies to artificial flowers of four colors (quadruple choice-test): yellow, pink, white, and red; their choices were assessed. In Experiment 2, we examined the ability of this butterfly species to associate colors with rewards while exploring if the spectral reflectance value of a flower color can slow or accelerate this behavioral response. Butterflies were first trained to be fed from artificial yellow flowers inserted in a feeder. These were later replaced by artificial flowers with a similar (blue) or very different (white) spectral reflectance range. Each preference test comprised a dual-choice test (yellow *vs* blue, yellow *vs* white).

**Results:**

Butterflies showed an innate strong preference for red flowers. Both the number of visits and the time spent probing these flowers were much greater than the pink, white, and yellow color flowers. Butterflies learn to associate colors with sugar rewards. They then learned the newly rewarded colors as quickly and proficiently as if the previously rewarded color was similar in spectral reflectance value; the opposite occurs if the newly rewarded color is very different than the previously rewarded color.

**Conclusions:**

Our findings suggest that common green-eyed white butterflies have good learning abilities. These capabilities may allow them to respond rapidly to different color stimulus.

## Introduction

The ability to interact with dynamic and unpredictable environments is crucial to an organism’s survival and fitness. For an animal pollinator, continually facing the ever-changing availability of their floral rewards as well as the ability to detect, learn, and switch among flowers is fundamental (Proctor, Yeo & Lack, 1996). Learning occurs when an animal interacts with the surrounding environment and adapts its behavior based on experience and innate sensory biases (Dukas, 2008; Matthews & Matthews, 2010). While locating nectar sources may seem like a relatively easy task for an insect—particularly in habitats containing a multitude of floral choices—research has shown that the decision-making process is not so simple (Chittka & Thomson, 2001). An insect must learn to navigate the morphology of a flower to extract nectar, but the morphology is different for each flowering plant species. As such, learning to associate floral cues with the presence of a reward could increase foraging efficiency if it allows the pollinator to locate a new flower of the same species and quickly extract the nectar. Floral color plays an essential role in this task (Trunschke et al., 2021). In contrast, innate preferences—which have been extensively investigated in flower-visiting social insects (Lunau & Maier, 1995; Kelber, Vorobyev & Osorio, 2003)—are believed to account for biases that aid in recognition, and possibly learning, of critical floral resources (Riffell et al., 2008).

Eusocial insects such as honey bees, stingless bees and bumble bees have been a major focus of research in the foraging behavior of insect pollinators. The ability to learn various stimuli such as color and odor has been explored in detail in the foraging context of these insects (Chittka & Thomson, 2001; Dyer et al., 2016). However, solitary insects such as non-eusocial bees and butterflies exhibit similar cognitive abilities as is seen in other hymenopterans; this behavior signifies their ability to learn and use floral cues for efficient foraging (Lewis, 1989; Goulson & Cory, 1993; Weiss, 1997; Goulson, Ollerton & Sluman, 1997; Blackiston, Briscoe & Weiss, 2011; Howard, Garcia & Dyer, 2021).

Innate color preferences have previously been shown in some butterfly species, and each family tends to display different color preferences. For example, in Nymphalidae, *Aglais urticae* (Scherer & Kolb, 1987a), *Argynnis paphia* (Ilse, 1928), *Inachis io* (Ilse, 1928), *Nymphalis xantomelas* (Honda, 1976), and *Vanessa indica* (Ômura & Honda, 2005) prefer yellow and blue colors. The Pieridae butterflies including *Gonepteryx rhamni* (Ilse, 1928), *Pieris brassicae* (Scherer & Kolb, 1987b), and *Pieris rapae* (Miyakawa, 1976) show a high preference for yellow, purple, and blue. Likewise, some Papilionidae species such as *Battus philenor* (Weiss, 1997), *Papilio demoleus* (Ilse & Vaidya, 1956), *Papilio machaon* (Ilse, 1928), and *Papilio troilus* (Swihart, 1970) share a preference for purple and blue while *Papilio xuthus* prefers yellow and red (Kinoshita, Shimada & Arikawa, 1999). This evidence suggests that closely related species have similar color preferences when foraging. These preferences might be attributed to the phylogenetic characteristics of their color vision (Briscoe et al., 2003).

Innate color preferences have been suggested to provide behavioral biases that aid in the initial location or recognition of flowers (Gumbert, 2000; Goyret et al., 2008). However, associative learning comes into play once a butterfly lands on a flower. In recent years, lepidopterans have received increasing attention for their ability to associate both preferred and non-preferred colors with a reward (*e.g*., Goyret & Kelber, 2011; Stewart, Kinoshita & Arikawa, 2015; Rodrigues, 2016; Dell’Aglio, Losada & Jiggins, 2016; Ramos, Rodríguez-Gironés & Rodrigues, 2017). For example, associative color learning in the context of feeding has been demonstrated in some well-studied species such as *Heliconius charitonius*, *Heliconius erato phyllis*, *Danaus plexippus*, and *Papilio xuthus* (see a review in Kinoshita, Stewart & Ômura, 2017). In nature, butterflies may experience unrewarding and rewarding flowers of different colors simultaneously at a given site. Thus, they must constantly make foraging decisions about which patches, plants, and flowers to visit. The uncertainty of these decisions in a novel environment can be reduced if an individual quickly associates a rewarding flower with its color and then discriminates non-rewarding flowers to avoid revisitation. Butterflies possess either tri- or tetrachromatic color vision based on a set of UV, blue, green and red receptors and sensitive to at least 600 nm (Koshitaka et al., 2008; Marshall & Arikawa, 2014), capabilities used to discriminate a rewarding flower, approach, land and take nectar from the flower (Koshitaka, Arikawa & Kinoshita, 2011). Although butterflies typically pollinate primarily yellow and pink flowers (Yurtsever, Okyar & Guler, 2010), they also feed from flowers of different colors throughout their ranges. They are flexible and move quickly to rewarding colors other than the above-mentioned colors once they are sampled. Thus, it’s crucial to know the spectrum reflectance characteristics of a surface butterflies perceive in order to understand how they perceive an environment. These may be documented in the form of spectral reflectance curves (*i.e*., plots of the percentage of incident radiation reflected at each wavelength across the region of the spectrum relevant to the visual system of the organism under study; Dorin et al., 2020). Photoreceptors in butterflies’ color vision transform captured light into signals that can be processed by the butterfly’s brain (Briscoe et al., 2010). Butterflies are particularly sensitive to color variation where surface reflectance changes rapidly in regions of overlapping photoreceptor sensitivity, allowing the butterfly’s brain to compare the signals from 6 to 15 types of photoreceptors (Arikawa, 2003; Chen et al., 2016). Here, we hypothesize that in floral colors most similar in terms of spectral reflectance curves, an insect pollinator should increase the speed/accuracy associated the presence of a reward (Chittka et al., 2003; Dyer & Chittka, 2004; Rodrigues, 2016). There should be a slower association in more distant floral colors.

Despite the potential importance of innate preference and learning for floral visitation, little is known about the generality of these behaviors in nonsocial insects such as the butterflies. In this study, we used experimental approaches to address basic questions about innate color preferences and learning abilities of the common green-eyed white butterfly (*Leptophobia aripa*, Boisduval, 1836) whilst foraging for artificial flowers of different colors. This insect is a multivoltine species with overlapping generations that specializes in the family Brassicaceae. It is an important pest of Brassica crops in Southeastern Mexico, Central America, and the Caribbean (CATIE/MIP, 1990). Here, we first tested innate preferences towards a specific floral color stimulus. Second, we asked whether *L. aripa* can also learn to associate a second color with a reward as rapidly as they do the first particularly as a function of the similarity in spectral reflectance of both colors.

## Materials & methods

### Study system and general procedures

From September to November 2019 (Experiment 1) and February to March 2020 (Experiment 2), we used virgin adults of the common green-eyed white butterfly (*Leptophobia aripa*) from a butterfly farm culture derived from eggs laid by females caught around the campus of Ixtacuixtla at the Universidad Autónoma de Tlaxcala, Tlaxcala, México (19°19′N, 98°20′W, 2240 m a.s.l.). The hatched larvae were fed on fresh cabbage leaves at ±26 °C under a 12 h:12 h light:dark cycle in 1,000-mL plastic transparent pots covered with lids with holes for air exchange (Laboratorio de crianza de Lepidoptera). Pupae were allowed to emerge in these boxes and monitored daily for emergence. All butterflies had similar ages (1–2 days) at the start of the experiments.

### Experiment 1: innate color preference

We conducted innate preference choice-tests to determine whether *L. aripa* butterflies had a strong innate preference for specific colors and then evaluated whether relative color preferences differed depending on the array of colors offered. The innate preference experiment was comprised of two phases: (1) The 5-min priming phase allowed butterflies to hand-feed on a black color artificial flower in order to enhance their association between artificial flower models and the presence of a sugar reward (*i.e*., by carefully unrolling the proboscis into 5 μl of 25% sugar solution); (2) The testing phase evaluated an individual’s preference for a specific flower color. The use of artificial black flowers to stimulate butterfly’s interest in foraging prior to the start of a given training or testing session is frequent in the literature (*e.g*., Swihart & Swihart, 1970; Rodrigues, Goodner & Weiss, 2010, Blackiston, Briscoe & Weiss, 2011; Rodrigues & Weiss, 2012; Cepero, Rosenwald & Weiss, 2015, de Oliveira, Trigo & Rodrigues, 2015; Rodrigues, 2016; Esmaile & Rodrigues, 2020). Previous studies have shown that butterflies do not visit artificial flowers of this color during foraging trials (*e.g*., Swihart & Swihart, 1970; Esmaile & Rodrigues, 2020). Thus, butterflies confronted with artificial black flowers will not be conditioned to this floral color, but only stimulated to search for food.

**Figure 1:**
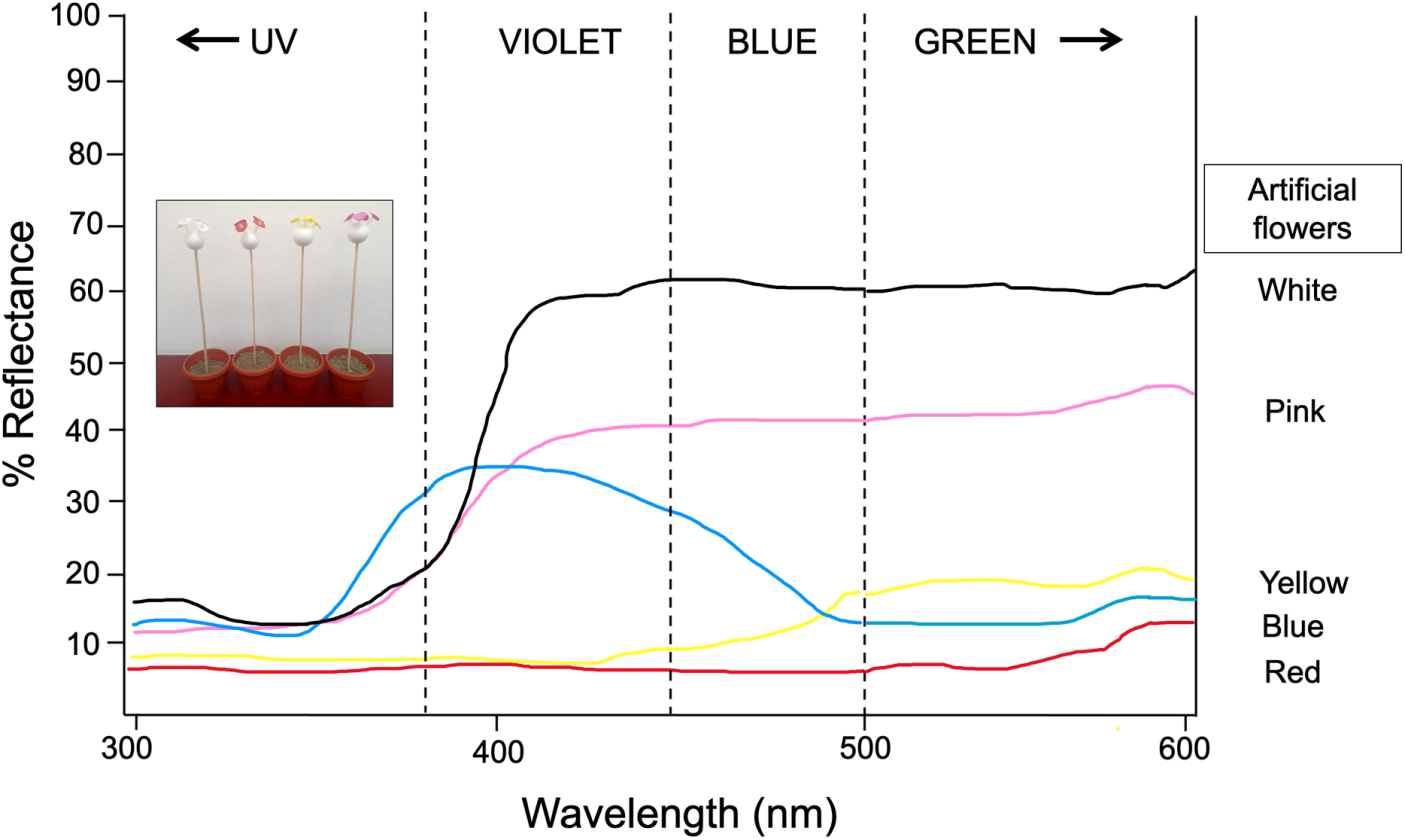
Spectral reflectance profiles for each colored artificial flower used in experiment 1 and 2. Measures obtained using a VARIAN-CARY 2415 spectrophotometer calibrated to measure reflected light in the range of 300–600 nm. Modified from Ornelas & Lara (2015). Photograph shows the experimental setup by using the artificial flower arrays. Photo credit: Carlos Lara.

Artificial flowers were made using conical plastic micropipette tips 58 mm length with simulated “petals” made of not glossy plastic materials (flagging tape) of different colors (white, yellow, pink, and red for Experiment 1 and blue por Experiment 2). Diffuse reflectance measurements of this plastic material were previously obtained by Ornelas & Lara (2015) using a VARIAN-CARY 2415 spectrophotometer calibrated to measure reflected light in the range of 300–600 nm. Spectral reflectance of flowers was taken at an angle of 45° to the measuring spot. A pellet of barium sulphate was used as a white standard and a black film can was used as a black standard. We mapped the reflectance spectrum obtained into Chittka’s hexagon model with a green leaf as background (Chittka, 1992) and then calculated the Euclidean distances between pairs of sample dots as the color dissimilarity between artificial petals by using the vismodel function of the package pavo (Maia et al., 2019). These calculations allowed a differential categorization of these colors in the Experiment 2, based in the maximum dissimilarity distance between white and the grey colors (*i.e*., blue and yellow). The color models used here differ in hue (the major wavelength reflected from a substrate) as well as in intensity (the amount of light reflected from surface at a given wavelength) (Fig. 1). We constructed four flower arrays: Each was made of four flowers of a specific color, which were inserted into small balls of Styrofoam, which in turn was inserted into a wooden stick (30 cm length). Each array was inserted into a flowerpot and placed inside a field collapsible cage (61 × 61 × 61 cm). Behavioral experiments were carried out in the cage illuminated with eight halogen lamps (300 W; Toshiba, Japan) hanging from the ceiling. The illumination does not emit ultraviolet light. Such UV suppressed illumination has been found not to affect the color choice behavior in some butterfly species such as *Papilio xuthus* (Kinoshita & Arikawa, 2000).

Thirty-two butterflies were trained and tested. After allowing a butterfly to feed on the black artificial flower for 5-min, butterflies were immediately released individually into the field cage to visit the artificial flowers for 30 min in a quadruple choice-test (white *vs* yellow *vs* pink *vs* red). The observer was located in front the cage (ca 0.5–1 m). We recorded the following for each focal butterfly: (1) the color of the first visited flower, (2) the number of visits on each color model, as well as (3) the time spent probing each model. A visit was defined as landing on a model from flight in which a probe occurred; a probe was defined as the unraveling of the proboscis and contact with the artificial flower. Observations were conducted from 11:00 to 14:00 h (peak of diurnal butterfly activity). After each trial was completed, the butterfly was removed from the cage and returned to the butterfly farm.

### Experiment 2: associative color learning

We next evaluated whether butterflies trained in captivity favor a particular floral color. Four artificial yellow flowers made as above were inserted into a transparent no colored plastic hummingbird feeder containing 250 mL nectar reward (25% sugar solution). We monitored whether animals change their foraging preference when a new nectar rewarded floral color replaced the previous four yellow color flowers. Thus, we used two new colors of flowers (blue and white) to further determine if the change in foraging preference is also affected by the similarity (*i.e*., blue color) or difference (*i.e*., white color) in the spectral reflectance curves relative to the yellow color.

Thirty newly emerged butterflies (following the same rearing protocol described in Experiment 1) were kept in the butterfly farm (6 × 8 × 2.30 m) and trained to feed from a nectar rewarded feeder containing four artificial yellow flowers for seven consecutive days. Immediately afterwards, for four continuous days and starting at 11:00 h, the elapsed times between each visit of the butterflies to the feeder—as well as the time spent probing each visited flower—were recorded for 30 consecutive minutes. At the end of the observation period, the four yellow flowers were replaced by four blue flowers in the feeder, and the aforementioned response variables were again recorded for 30 more minutes. Thus, the flower replacement was repeated four consecutive days, and during each test phase the feeder was refilled. This protocol was repeated immediately after completion, but now the four yellow flowers were replaced with four white flowers. With this experimental design and dual-choice tests (yellow *vs* blue, yellow *vs* white), we expect that the butterflies changed their color preferences with respect to the presence of a reward, but also in relation to the similarity or difference in the spectral reflectance curves of the new rewarded color relative to the previous visited floral color. At 500–600 nm, artificial yellow and blue flowers have similar low reflectance (~15–20%), but white flowers have a higher reflectance peak (~60%) (Fig. 1). We expected the butterflies to visit the blue flowers faster and with greater frequency and duration because they are closer in reflectance to the yellow color initially rewarded. In contrast, the spectral reflectance curve of white artificial flowers is very far from the spectral curve of yellow (and even blue) flowers, therefore they should take much longer to be visited and with less frequency and duration of visits. The experiment was performed under natural daylight illumination coming from windows as well as a central skylight.

### Statistical analysis

In Experiment 1, we observed the immediate response of newly emerged butterflies to four floral colors as a proxy for innate color preference. Therefore, the color of the first flower visited was the nominal response variable. We use the G-test of goodness-of-fit (Zar, 1999). This statistic is useful when there is a nominal variable with two or more values, *i.e*., the four floral colors (white, pink, red and yellow). This test compares the number (or proportion) of visits recorded in each color against the expected number (or proportion) of visits. In this case, the null hypothesis was that the butterflies do not show a preference for any of the four floral colors; therefore, the visit ratio should be 1/4 = 0.25 per floral color. On the contrary, if there is a preference for a color then the proportions must be different from what is expected. The equation used for this statistic is: G = 2Σ (O × ln (O × E)), where O is the observed visit, E is the expected visit, and ln is the natural logarithm. Then, the effects of flower color on the number of visits and the time spent probing each visited flower were analyzed through generalized linear model (GLM) procedures (Poisson distribution, log-link function) in R version 3.6.0 (R Development Core Team, 2019). In the model, floral color was treated as a fixed factor, and the number of visits and their duration (*i.e*., duration per visit) were response variables. A Tukey post-hoc test was used for multiple comparisons among pairs of means.

In Experiment 2, we used survival analysis (“time failure analysis”) in R (R Development Core Team, 2019) to analyze differences in butterfly arrival times to feeders in both trials (yellow flowers-blue flowers, yellow flowers-white flowers). This statistical analysis uses censored data points in which an event is not observed because the study ended before the event could have happened to some individuals under observation. If an event occurred for a given artificial flower, then it became uncensored data. If it never occurred, then it became censored data (Muenchow, 1986). We used the Kaplan–Meier product-limit nonparametric method to compute the probability that butterflies had not yet visited an artificial flower in the arrangement 30 min after the start of observation. The log-rank statistic (Mante–Cox) tested for differences between trials. Differences in the number and duration of visits to the flowers of the different colors evaluated were analyzed using the Kruskal–Wallis non-parametric ANOVA and Dunn’s post-hoc pairwise comparison tests in R (R Development Core Team, 2019).

## Results

### Innate color choices

**Figure 2:**
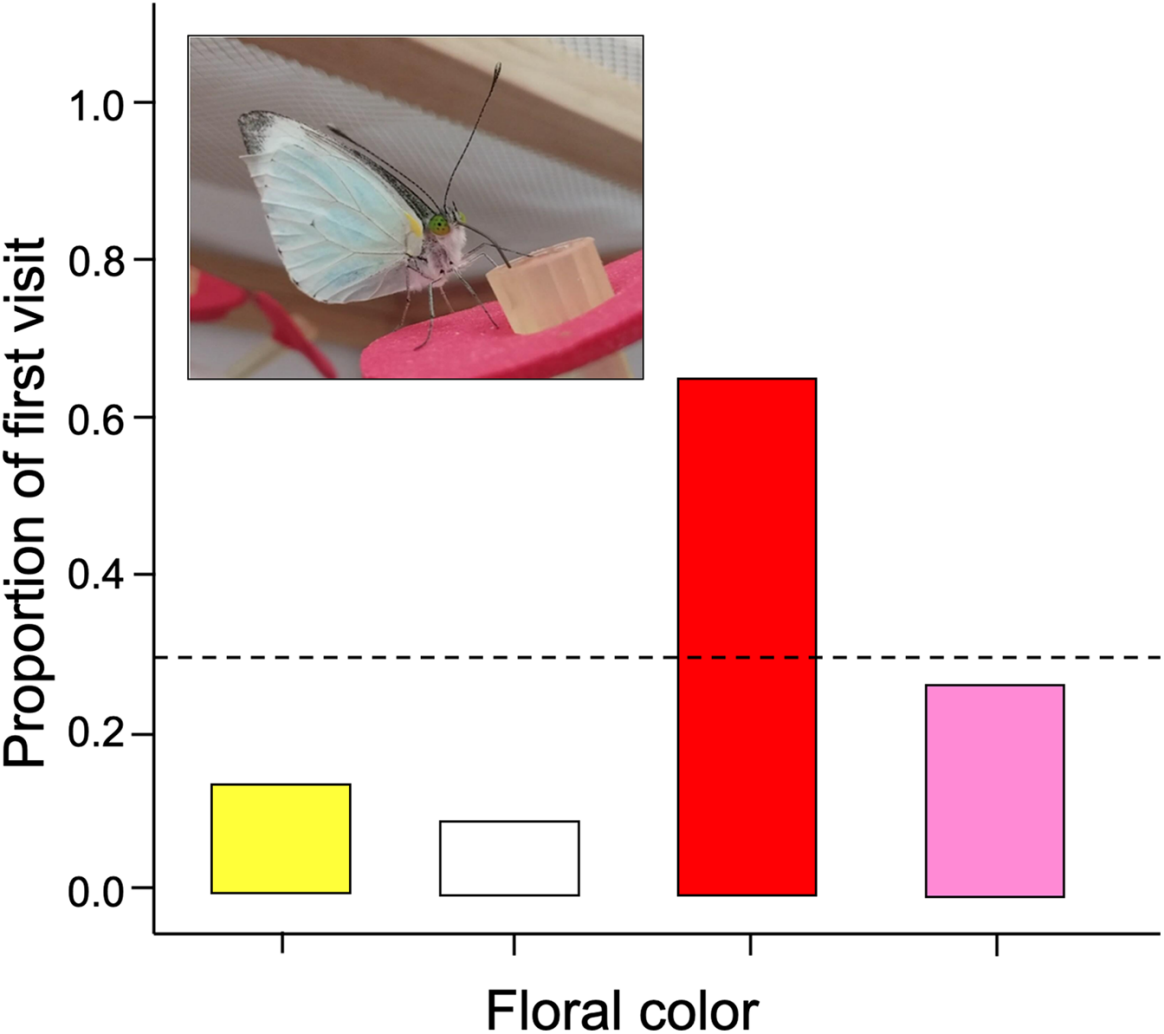
The initial color probed by naïve *Leptophobia aripa* butterflies faced to four artificial flower arrays. Visits showed significant deviations from random choice, with red being the most strongly preferred and white the least preferred color. Photograph shows a butterfly feeding from a red colored artificial flower. Photo credit: Carlos Lara.

We found that naive butterflies faced with four different colors tend to visit red flowers more frequently than expected (G-test of goodness of fit; G = 29.19; df = 3; *p* = 0.0006). Therefore, the null hypothesis is rejected because there are significantly more first visits to red flowers with respect to the other colors (Fig. 2).

**Figure 3:**
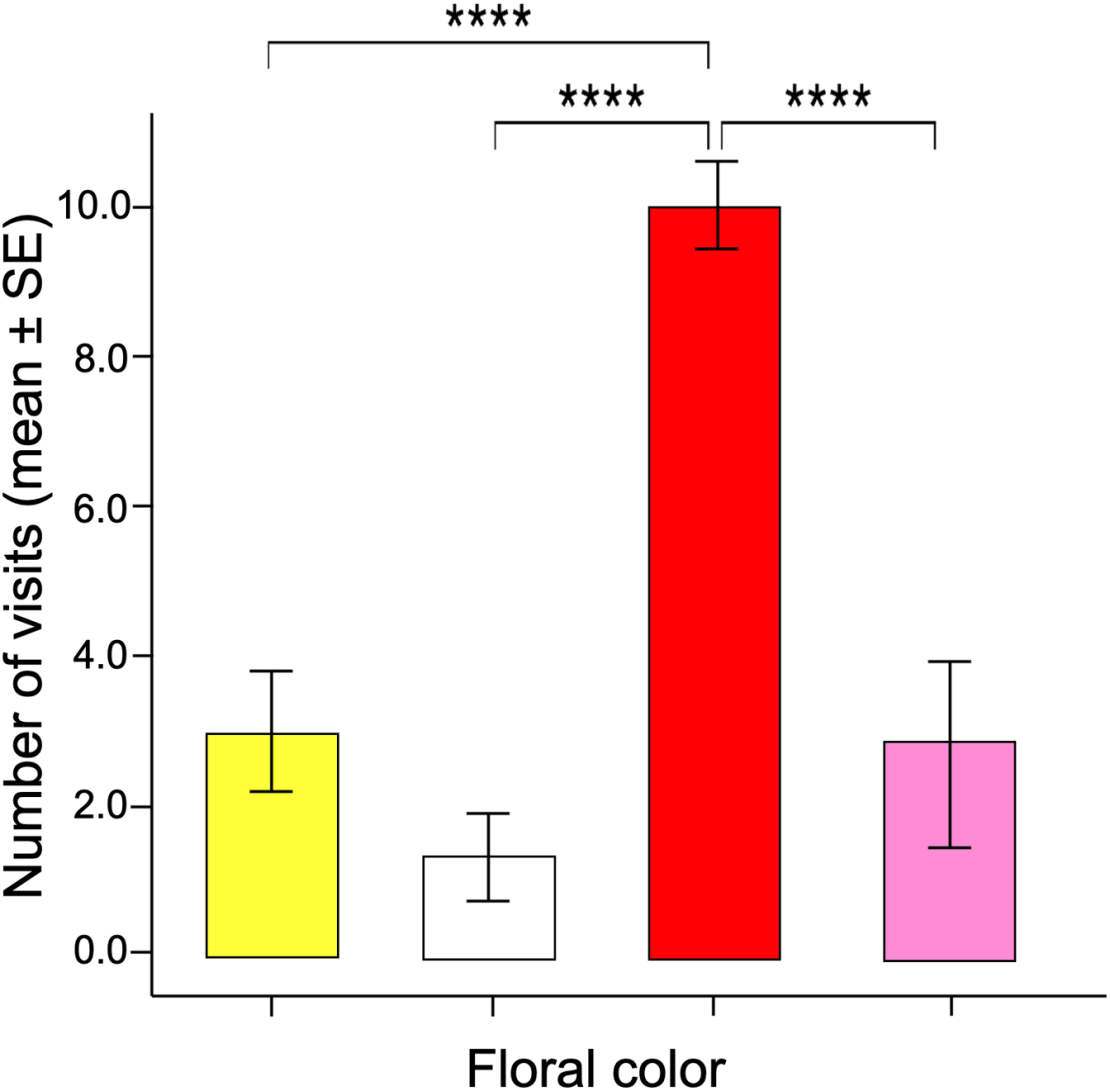
Innate color preference. When the naïve butterflies were confronted with the four colors in the artificial arrays, they showed an overwhelming number of visits to the red flowers compared to the other floral colors. Four asterisks (****) indicate highly significant difference (*p* < 0.00001).

The number of visits made over 30 min by each butterfly varied between the different facing colors according to the GLM model (ANOVA; χ^2^ = 17.96; df = 3; *p* < 0.0001). The red flowers received significantly more visits than the other floral colors in the arrangement (Fig. 3). Post-hoc mean contrasts (Tukey method) showed no significant differences between the number of visits to pink, white, and yellow flowers.

**Figure 4:**
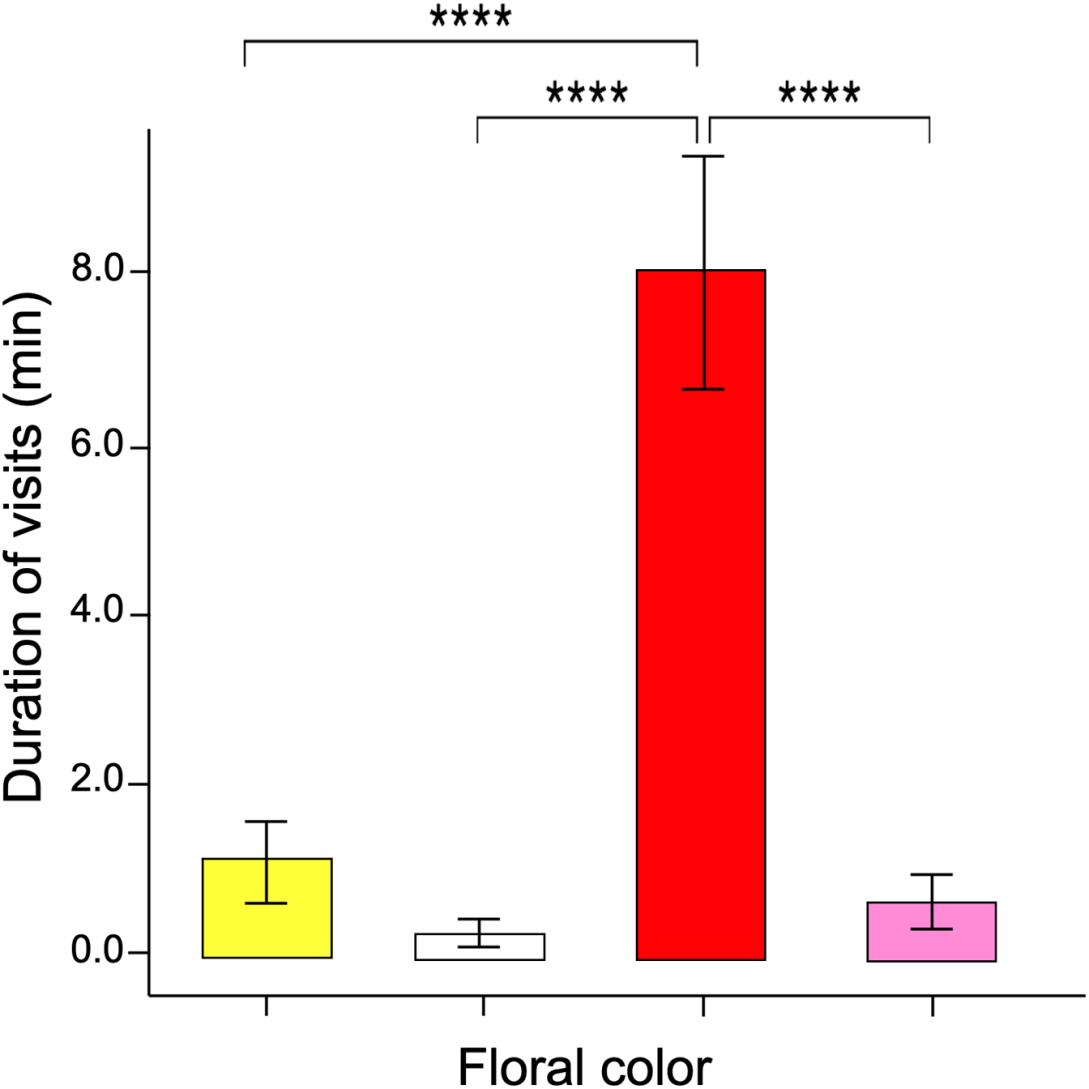
Duration of flower visits. The time spent probing the red flowers was much greater than that shown in the other flower colors. Four asterisks (****) indicate highly significant difference (*p* < 0.00001).

The GLM data tested differences in the time spent probing each color flower per visit (*i.e*., duration per visit). The results showed that visits to red flowers were longer than the other colors (ANOVA; χ^2^ = 3.16; df = 3; *p* = 0.032, Fig. 4). The post-hoc tests showed no statistically significant differences when comparing the duration of visits by butterflies to pink, yellow, and white flowers.

### Associative color learning

**Figure 5:**
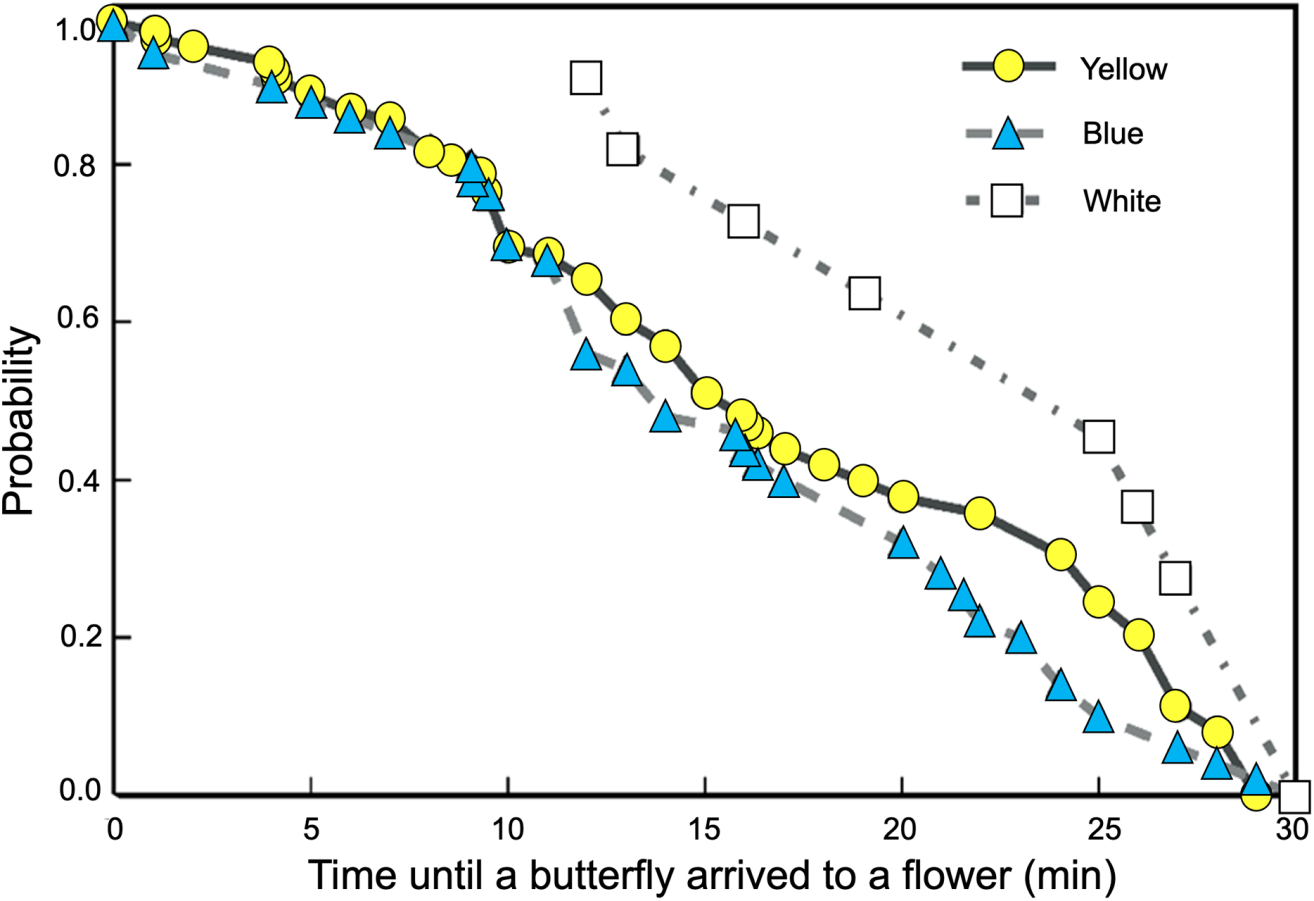
Butterflies learned to associate colored artificial flowers with a sucrose reward. The waiting times for visits to flowers of closer colors in spectral reflectance, such as yellow and blue, were not significantly different; but the time elapsed until the start of a visit significantly increases in white flowers.

We found significant differences between the probability curves of butterflies visiting feeders as differences in spectral reflectance of the rewarded flowers increased (Logrank–Mantel Cox: χ^2^ = 104.26, df = 2, *p* < 0.001). The waiting times for visits to flowers of closer colors in spectral reflectance, such as yellow and blue, were not significantly different (Logrank–Mantel Cox: χ^2^ = 0.91, df = 2, *p* = 0.72). However, when the white flowers were placed in the feeders, the time elapsed until the start of the butterfly visits increased significantly more compared to the yellow (Logrank–Mantel Cox: χ^2^ = 15.34, df = 1, *p* < 0.01) and blue flowers (Logrank–Mantel Cox: χ^2^ = 14.24, df = 1, *p* < 0.01; Fig. 5).

**Figure 6:**
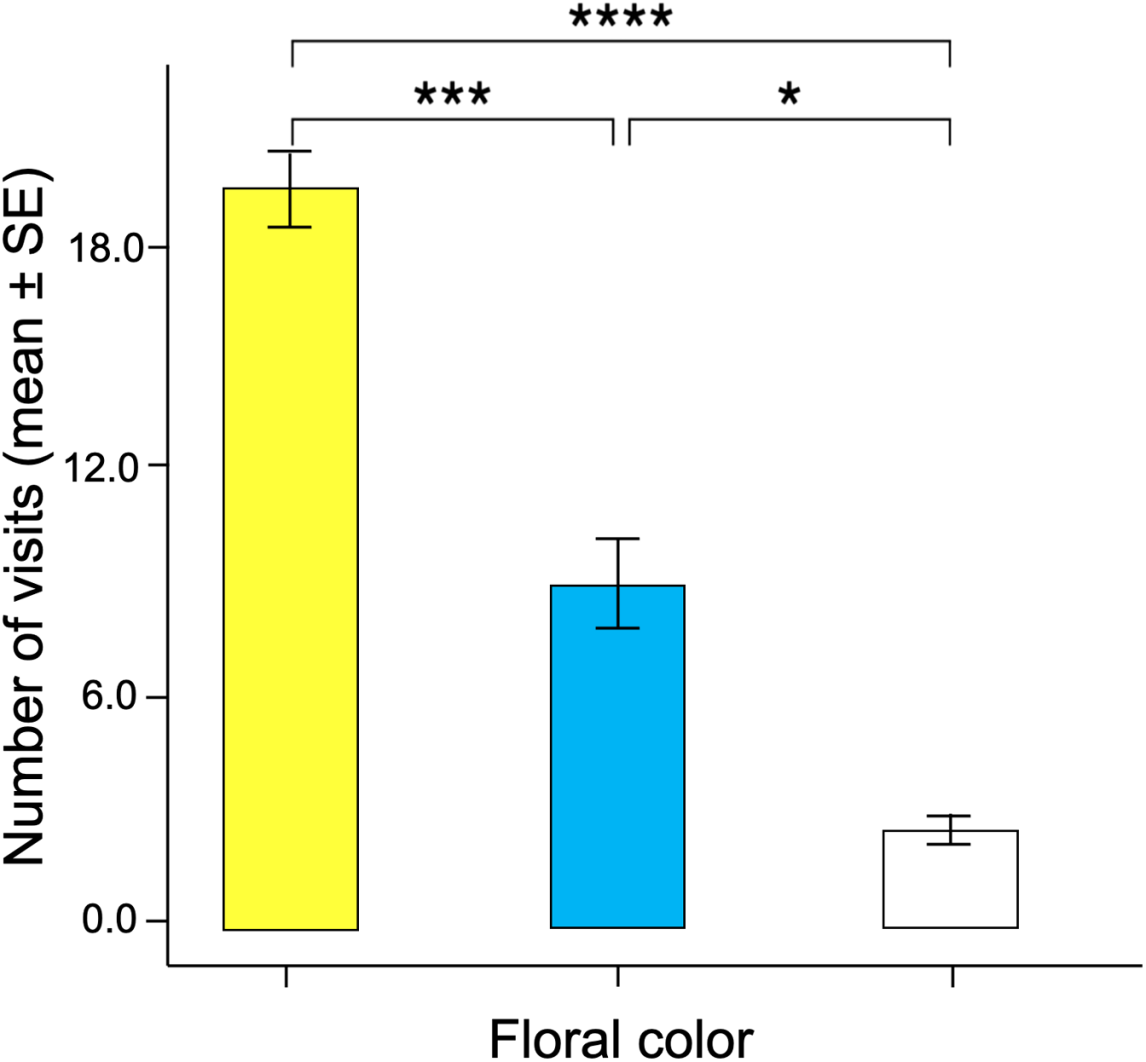
Associative color learning. When butterflies associated color with reward, they visited yellow flowers significantly more than the other floral colors, with white flowers being the least visited. An asterisk (*), three asterisks (***) and four asterisks (****) indicates statistical significance (*p* < 0.01, *p* < 0.0001 and *p* < 0.00001, respectively).

**Figure 7:**
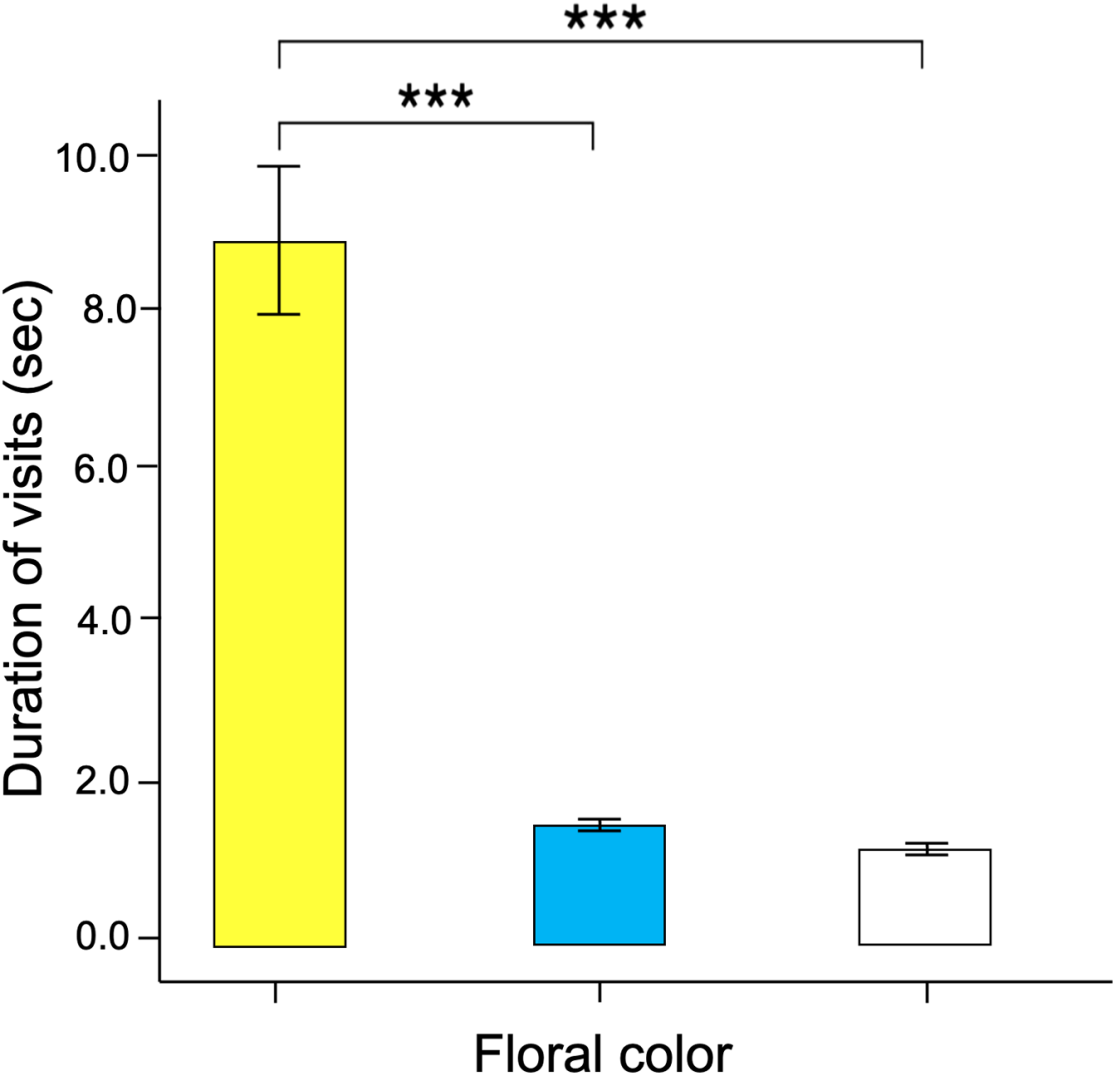
The time spent probing each training color statistically differ across colors. Butterfly visits to yellow flowers were longer than those made to blue and white flowers. Three asterisks (***) indicate highly significant difference (*p* < 0.0001).

Significant differences were found in the number of visits made by the butterflies to the three floral colors (Kruskal–Wallis test; H = 66.5; df = 2; *p* < 0.001; Fig. 6). Yellow flowers received a higher number of visits than blue flowers (Dunn’s *post hoc* tests; Q = 3.78, *p* < 0.0001) and white flowers (Q = 4.12, *p* < 0.00001). However, white flowers received even fewer visits than blue flowers (Q = 2.98, *p* < 0.01). Finally, the duration of the visits made to each of the floral colors faced by the butterflies was statistically different (Kruskal–Wallis test; H = 66.5; df = 2; *p* < 0.001; Fig. 7). The butterflies made significantly longer visits to yellow flowers than to blue (Dunn’s *post hoc* tests; Q = 3.98; *p* < 0.00001) and white colored flowers (Q = 3.02; *p* < 0.01). However, the duration of the visits made to blue flowers was not statistically different than the duration of visits to white flowers (Q = 1.79; *p* = 0.462).

## Discussion

We found that the common green-eyed white butterflies (*Leptophobia aripa*) preferentially visited red flowers. In Experiment 1, red flowers preferentially received the first visits and were visited with greater frequency and duration than the other floral colors. In Experiment 2, we demonstrated that butterflies previously trained to visit a certain floral color (yellow) showed flexibility when visiting a new rewarded color (blue or white). However, this flexibility seems to be affected by the reflectance curve value of the colors in such a way that a color similar in reflectance to the one previously detected promotes fast visits; a color far from the initially visited value delays the start of visits. Although they had choice flexibility, the butterflies did show some preference to the previously detected color (yellow), which was seen in a greater number and duration of their visits.

The innate preference for colors, shapes, and odors has been interpreted as a way of providing behavioral biases that helps insects to recognize and probably learn appropriate cues in an environment (Gould, 1984; Gould & Marler, 1984; Giurfa et al., 1995; Lunau & Maier, 1995). In butterflies, the innate color preferences reported to date differs between genera and even between species. For example, newly emerged *Battus philenor* butterflies showed a spontaneous preference for yellow both in experiments using paper circles or true yellow flowers (Weiss, 1997). However, other studies using different species of butterflies, even from the same family (Papilionidae), report very different preferences (*e.g*., Ilse, 1928; Ilse & Vaidya, 1956; Swihart, 1970; Kinoshita, Shimada & Arikawa, 1999).

For instance, *Papilio machaon* and *P. demoleus* preferred blue and purple papers (Ilse, 1928; Ilse & Vaidya, 1956), but *P. troilus* preferred blue papers with a lesser preference for orange (Swihart, 1970). Interestingly, among butterflies in the Nymphalidae family, the innate preference for specific colors also varies between species. *Heliconius charitonius* showed a preference for orange/red flowers followed by blue/green colors (Swihart & Swihart, 1970), while *Vanessa io* and *V. urticae* similarly preferred yellow and blue flowers, and *V. polychlorus* showed a strong preference for yellow flowers (Ilse, 1928). Pieridae butterflies are no exception and variable responses in innate color preference have also been reported. For example, in spontaneous-choice experiments where the red color was included, *Gonepteryx rhamni* (Ilse, 1928), *Pieris brassicae* (Scherer & Kolb, 1987b), and *Pieris rapae* (Miyakawa, 1976; Kandori & Ohsaki, 1996), showed a high preference for yellow, purple, and blue, respectively. Thus, our study represents the first record of a butterfly from this family showing an innate preference for red. However, in our Experiment 1 we cannot rule out possible effects of the training of the butterflies on black targets for their subsequent color choice. Our finding could be a more direct associative mechanism that red is close to black in overall intensity. Thus, to control for intensity it would be of high value to prime butterflies to a high intensity and spectrally neutral stimulus (*e.g*., aluminium for reflects 300–700 nm radiation equally and at much higher levels). Future studies should replicate our findings if a group of the butterflies are primed to a high intensity stimulus.

Butterfly-pollinated flowers can have a wide variety of colors including yellow, orange, pink, red, purple, and blue (Kevan & Baker, 1983). However, the different color preferences among ecologically similar butterfly taxa are unlikely to be close to actual differences in the prevalence of appropriate flower colors. The ability to learn can make the identity of the naturally preferred color less critical. For example, Yurtsever, Okyar & Guler (2010) studied the flower color preferences of 66 plant species visited by 43 Lepidopteran species (most from the Nymphalidae family) and found that yellow flowers were the most visited. Interestingly, none of the butterfly species reported in that study visited red flowers. A possible explanation for this lack of attraction for the red color may be supported by aspects other than ecological ones. Indeed, some butterfly species in the Nymphalidae family do not have red-sensitive photoreceptors (Briscoe & Bernard, 2005). In contrast, other studies have shown that butterflies from other families can distinguish red from colors such as green, blue and yellow (Kevan & Baker, 1983; Scherer & Kolb, 1987b; Kelber & Pfaff, 1999). In this sense, most studies supporting vision in the red by butterflies have been experimental and have involved learning behavior. For instance, the pioneering work of Kühn & Ilse (1925) used *Gonopteryx rhamni* and *Pieris brassicae* (Pieridae) and showed the preference for red. In a way, these preferences can change according to the circumstances of availability of rewards in the environment as demonstrated by Goulson & Cory (1993) who showed that the preference for red or blue flowers is not inherent in *Pieris napi*; rather, butterflies can be flexible in their preferences depending on the reward presence in a certain color. Thus, it is clear that butterflies exhibit a very wide visual range, and color is an important cue to visit a wide variety of plants in nature where innate preferences can be shaped by learning.

Newly emerged flower visitors may exhibit color preferences prior to individual flower experience. Understanding the innate color preferences of flower visitors requires detailed analysis because color is a multi-signal stimulus in the first instance (*i.e*., not only elemental factors that influence human perception such as color hue and intensity). For example, the contrast between a stimulus and its background mediated solely by the green photoreceptor are also considered to be important factors enabling the visual perception of flowers by pollinators (van der Kooi et al., 2019). Likewise, some flying pollinators (*e.g*., honeybees and butterflies) also use achromatic intensity contrast especially when the targets are small (Koshitaka, Arikawa & Kinoshita, 2011; Dyer, Spaethe & Prack, 2008). Additionally, different components of the flower-visitor-subjective color appearance of flowers must be distinguished (*e.g*., hue, saturation, brightness, and other potentially visible characteristics) for correctly identifying possible selection targets, as demonstrated by laboratory studies (Trunschke et al., 2021). In addition, flower visits include a sequence of behavioral reactions, which can be driven by innate bias. Behavioral reactions such as distant focus, close-range flower orientation, landing, and mouthpart extension can be triggered by color stimuli (Lunau & Maier, 1995). Physiological limitations of spectral sensitivity, neurosensory filters, and different abilities of animals to make use of visual information such as brightness perception, wavelength-specific behavior, and color vision shape color preferences (Menzel & Shmida, 1993). In addition to these receptor-based factors, there are restrictions on flower coloration due to emitter-based factors such as the absorption properties of flower pigments and the dual role of flower colors that trigger innate and learned behavior (Kevan & Lane, 1985). Representations of the spectral reflection of colored objects activate innate color preferences and provide an objective measure of color stimuli (Lunau & Maier, 1995), thus allowing analysis of color preferences in both natural and experimental conditions (as in this study). The innate preference for the red color of *L. aripa* shown here represents an honest indicator in the search for food—especially if we consider that the females of this species usually tend to oviposit in Mexican cress (*Tropaeolum majus* L.), a ruderal plant with red-orange flowers, where adults feed mainly. In this way, after the formation of the imago and once they emerge as adults, the butterflies have a high probability of facing flowers of this color and thus have a sensory bias to visiting them. This preference could be later modified by the availability of nectar in the environment. Whether the butterflies would prefer a red-orange color similar to the flowers of their food plant over a red color deserves to be analyzed in future studies.

Learning should evolve in unpredictable environments in such a way that behavioral fixed patterns in an individual are appropriate, but not so unpredictable that the individual cannot behaviorally follow their change (Stephens, 1993). Learning plays an important role in food acquisition for a butterfly and these insects may learn to associate floral cues with the presence of a reward while increasing their foraging efficiency (Weiss, 1997). Although previous studies suggest that Pierid butterflies can discriminate and find nectar-providing flowers by their colors (*e.g*., Kandori & Ohsaki, 1996; Kelber, 2001), they do not conclusively demonstrate color learning (but see Arikawa et al., 2021). In fact, there are earlies examples suggesting a small ability of pierid butterflies to learn colors. For example, Scherer & Kolb (1987b) used spontaneous-choice experiments to show that the butterfly *Pieris brassicae* cannot be trained to give a feeding response to monochromatic light stimuli. Likewise, Goulson & Cory (1993) showed that *Pieris napi* butterflies confronted to artificial flowers of two colors exhibited flower constancy, with a strong preference for one color of the other (even if flowers were empty). In our study, *L. aripa* butterflies trained to visit yellow flowers in a feeder quickly changed to visit blue flowers when they replaced them. Although it took them a long time to start their visits, the butterflies also associate white flowers with the presence of a reward. Previous studies in butterflies have found similar results. For example, Lewis & Lipani (1990) showed that *Pieris rapae* quickly learned to associate the blue or yellow colors with a sugar reward after a single exposure—this learning rate is comparable to that observed in honey bees (Menzel, 1993). In another study, Weiss (1997) showed that *Battus pilenor* butterflies trained to visit yellow flowers changed their color preferences after only 10 visits to unrewarded yellow flowers. This changed towards visiting red (rewarded) flowers. *L. aripa* butterflies can learn to associate blue and white flowers with the presence of a reward in trials that lasted for 30 min of exposure after replacing the yellow flowers. This led to more experience in training feeder and therefore an even greater preference for the yellow flowers (*i.e*., butterflies made more visits and spent more time probing each visited flower). A similar increase in preference with continued reward experience has previously been reported in other insects both in color and in other learning stimuli. For example, *Agraulis vanillae* butterflies also become more discriminatory in favor of rewarded yellow flowers (Weiss, 1995). Naive bumblebees, *Bombus* spp., learned to visit morphologically complex flowers “correctly” in about 10 visits; although the handling times did not approach those of experienced bees until 30 to 60 flowers were visited (Laverty, 1980, 1994).

Butterflies’ choice of flower color in nature likely reflects an interplay between innate and learned color preferences. Swihart & Swihart (1970) concluded that the fundamental instinctual patterns of flower-seeking behavior in the *Heliconius charitonius* butterfly are not modified by conditioning but rather coexist with learned patterns. They showed that butterflies trained in one of their innately preferred colors (orange/red or blue/blue-green) showed a strong choice for that color, but those trained in a different color generally visited both the innate color and the conditioned color. A similar combination of innate and learned preferences was reported for *Battus philenor* in flower color choice (Weiss, 1997). Here we showed (Experiment 1) that *L. aripa* has an innate preference for the red color, and at 500–600 nm the spectral reflectance curve of artificial flowers of this color (~7%) is very close to yellow (~15%) and even blue (~20%) especially if we compare it with white artificial flowers (~60%). We found a similar combination of innate and learned preference (Experiment 2) where flowers whose reflectance value is close to the innately preferred ones are quickly visited, but those far from these values are discriminated or are visited late. On the contrary, the presence of nectar in white flowers (the most different in spectral reflectance curve value) seems to counteract the innate preference for certain reflectance values (red, yellow, blue). Our findings provide evidence that common green-eyed white butterflies have good learning abilities to respond rapidly to changing color stimulus.

Plants attract pollinators by displaying flowers of different colors (Faegri & Van der Pijl, 1979; Kevan & Baker, 1983). The role of flower colors in pollination has been studied in many experiments providing valuable information on the complex nature of plant-pollinator interactions (Menzel & Shmida, 1993*; Hempel de Ibarra, Vorobyev & Menzel, 2014*). Much of what we have learned about butterfly color preference comes from laboratory studies usually using artificial flowers (Kelber & Pfaff, 1999; Weiss, 1997; Kinoshita, Shimada & Arikawa, 1999; Weiss & Papaj, 2003; Blackiston, Briscoe & Weiss, 2011). Therefore, despite the information we have about the color preference of butterflies, we know little about how these behaviors translate into natural systems. These laboratory studies suggest that many butterflies display innate color preferences as well as learned associations between colors and nectar rewards. However, much remains to be investigated about the biological significance of these learning abilities in butterflies. Finally, understanding how pollinators make foraging decisions can provide insight into the reproductive and evolutionary success of both plants and pollinators. This is an interesting area of research from theoretical and applied perspectives.

## Conclusions

Our results showed that green-eyed white butterflies have an innate strong preference for red flowers. In our experiments, both the number of visits and the time spent probing these flowers were much greater than the pink, white, and yellow color flowers. Likewise, these butterflies learn to associate colors with sugar rewards, and then they can change to visit the newly rewarded colors as quickly and proficiently as if the previously rewarded color. This change is faster if the flower color is similar in spectral reflectance value; but the opposite occurs if the newly rewarded color is very different than the previously rewarded color. Our findings suggest that this butterfly species have good learning abilities. These capabilities may allow them to respond rapidly to different color stimulus.

## Supplemental Information

10.7717/peerj.12567/supp-1 Supplemental Information 1 Experiment 1. Innate color preferences. First visits, number of visits and duration of visits by naive butterflies faced to artificial floral arrays of different colors.

10.7717/peerj.12567/supp-2 Supplemental Information 2 Experiment 2. Associative color learning. Data showing the time until a butterfly arrived to a flower (yellow, blue or white). The frequency of visits (number of visits) and the duration of visits were obtained from these censored data.

## Additional Information and Declarations

### Data Availability

The following information was supplied regarding data availability: Our butterfly behavioral data are available in the Supplemental Files.
